# Changes to Intermediary Metabolites in Sporadic and *LRRK2* Parkinson's Disease Demonstrated by Proton Magnetic Resonance Spectroscopy

**DOI:** 10.1155/2015/264896

**Published:** 2015-08-18

**Authors:** Jan O. Aasly, Oddbjørn Sæther, Krisztina K. Johansen, Tone F. Bathen, Guro F. Giskeødegård, Linda R. White

**Affiliations:** ^1^Department of Neuroscience, Faculty of Medicine, Norwegian University of Science and Technology (NTNU), 7491 Trondheim, Norway; ^2^Department of Neurology, St. Olav's Hospital, University Hospital of Trondheim, 7006 Trondheim, Norway; ^3^Clinic of Radiology and Nuclear Medicine, St. Olav's Hospital, University Hospital of Trondheim, 7006 Trondheim, Norway; ^4^Department of Neurology, Akershus University Hospital, 1478 Lørenskog, Norway; ^5^Department of Circulation and Medical Imaging, Faculty of Medicine, Norwegian University of Science and Technology (NTNU), 7491 Trondheim, Norway

## Abstract

*Background*. Parkinson's disease (PD) remains a clinical diagnosis and biomarkers are needed to detect the disease as early as possible. Genetically determined PD provides an opportunity for studying metabolic differences in connection with disease development. *Objectives*. To study the levels of intermediary metabolites in cerebrospinal fluid (CSF) from patients with PD, either of sporadic type or in carriers of the *LRRK2* p.G2019S mutation. *Methods*. Results from patients with sporadic PD or *LRRK2*-PD were compared with asymptomatic *LRRK2* mutation carriers and healthy control individuals. CSF was analysed by proton MR spectroscopy (^1^H-MRS) giving reliable results for 16 intermediary metabolites. Partial least squares discriminant analysis (PLS-DA) was applied to study group differences. *Results*. PLS-DA distinguished PD patients from healthy individuals based on the metabolites identified in CSF, with 2-hydroxybutyrate, glutamine, and dimethyl sulphone largely contributing to the separations. *Conclusion*. Speculatively, all three metabolites could alter concentration in response to metabolic changes connected with neurodegeneration; glutamine as a means of removing excess nitrogen from brain, dimethyl sulphone as an anti-inflammatory agent, and 2-hydroxybutyrate in connection with altered glutathione metabolism. Potentially, ^1^H-MRS is a promising tool for identifying novel biomarkers for PD.

## 1. Introduction

Parkinson's disease (PD) is the second most prevalent neurodegenerative disease among the elderly, affecting approximately 1% over the age of 65 and around 5% by the age of 85 [1]. The disease is characterised by resting tremor, rigidity, and bradykinesia and can currently neither be prevented nor be cured. Control of disease symptoms can usually be achieved by dopamine replacement therapy, at least for some years. During the last two decades a number of genetic causes of PD have been discovered [2, 3]. While accounting for only a small proportion of all PD cases, the involvement of specific genes has provided novel insight into the mechanisms behind the pathophysiology of PD. Of the various genes hitherto identified, pathogenic mutations in the gene encoding leucine-rich repeat kinase 2 (*LRRK2*) at the* Park8* locus are amongst the most common [4], with the* 6055G>A* mutation resulting in a p.G2019S substitution in Lrrk2. The mutation is autosomal dominant and accounts for 2-3% of all PD cases in central Norway [5] but is clinically indistinguishable from sporadic PD.

The identification of genetically determined PD provides an opportunity for studying metabolic differences in connection with the development of disease. This is particularly important as dopamine turnover has been found to be elevated [6], as well as alterations to A*β*42 and tau protein levels [7] in asymptomatic* LRRK2* mutation carriers at risk of PD. Several studies employing singleplex or multiplex analysis have examined proteins known to be affected in genetic PD, or used as biomarkers in Alzheimer's disease, as potential biomarkers in PD [7–9]. Reliable biomarkers for PD remain to be identified however [10, 11]. We have previously shown that metabolomic profiling of plasma samples using high performance liquid chromatography coupled with electrochemical coulometric array detection (LCECA) distinguished patients with sporadic PD from those with* LRRK2*-related PD (*LRRK2*-PD) and even separated asymptomatic mutation carriers from family members without the mutation [12]. Trupp et al. have recently used metabolomics to demonstrate differences in a cluster of small metabolites in CSF between patients with PD and healthy control individuals [13]. In the present study we wished to examine the metabolomic profile of CSF in connection with sporadic PD and* LRRK2*-PD. The composition of CSF is determined partly by metabolic activity in the brain, as well as by changes in the composition of plasma. As opposed to the blood-brain and blood-CSF barriers, where penetration of substances like amino acids, glucose, or monocarboxylic acids requires carrier-mediated transport mechanisms, the brain-CSF barrier permits less restricted exchange of such molecules between CSF and the brain interstitial fluid [14–16]. Such differences may provide insight into (patho)physiological modifications of brain metabolism prior to the onset of symptoms in PD.

The present study employed proton magnetic resonance spectroscopy (^1^H-MRS), a technique that is now comparatively common in the clinical setting, to examine metabolite levels in CSF from* LRRK2*-PD patients and from patients without any known genetic cause for their PD (sporadic PD). The results were compared with metabolite concentrations in CSF from healthy (asymptomatic) individuals bearing the* LRRK2* p.G2019S mutation and from healthy individuals with no known movement disorder (controls).

## 2. Materials and Methods

All individuals in this study were ethnic Norwegians and provided written, informed consent. Study protocols for the samples were approved by the Regional Committee for Medical Research Ethics in Central Norway and the biobanks comply with Norwegian law. The patient cohort consisted of 27 PD patients (10 with* LRRK2*-PD), 11 healthy* LRRK2* mutation carriers, and 19 controls healthy for their age. Demographic data of the study participants are described in Table 1. All PD patients have been followed up by a movement disorder specialist (Jan O. Aasly) at the Department of Neurology, St. Olav's Hospital (Trondheim University Hospital), Trondheim, Norway. All participants were interviewed including the medical and family history, and a clinical and neurological examination was performed. A clinical diagnosis of PD required the presence of at least two of the three cardinal signs (resting tremor, bradykinesia, and rigidity), improvement with adequate dopaminergic therapy, and absence of atypical features or other causes of parkinsonism, consistent with a diagnosis of possible or probable PD according to the criteria proposed by Gelb and coworkers [17]. Cognitive impairment was assessed with the Minimental Score Examination (MMSE). Only one patient with* LRRK2*-PD and two patients with sporadic PD showed signs of mild cognitive impairment (MMSE 22–24). All patients were using anti-Parkinson medication (L-DOPA with or without dopamine agonists) as shown in Table 2, which was adjusted to a levodopa equivalent daily dose (LEDD) as used by Zibetti and coworkers [18] (Table 1). The control group was recruited from the outpatient clinic, from patients suspected of neurological conditions but where none was subsequently found. Six of these individuals took regular therapy: thyroxine replacement (3), hormone replacement therapy (oestrogen) (3), arrhythmia (1), and hypertension (1). No regular medication was recorded for the remaining controls. None of the group returned to the hospital for any reason during the next two years.

Genetic testing was performed as described previously [19]. Patients with sporadic PD did not carry any* LRRK2* mutation, whereas patients in the* LRRK2*-PD group were all found to be heterozygous carriers of the* LRRK2* p.G2019S mutation. Asymptomatic family members who volunteered were included if they tested positively for* LRRK2* p.G2019S. None of these individuals was attending the hospital, none demonstrated any premotor symptoms, and they did not take regular medication. All participants were informed that they would not be given any results regarding their genetic status, and only one coauthor (Jan O. Aasly) is aware of this status. Blood samples for genetic testing of patients in the control group were not available, though a series of over 1000 control blood samples from Norway has so far failed to find any sporadic* LRRK2* p.G2019S carriers.

Lumbar puncture was carried out on all participants during the morning (8–10 am) at the level of the lower lumbar spinous processes, mostly L4/L5. CSF samples were obtained immediately after the lumbar puncture, placed in ice-water, and stored at −80°C. Aliquots of CSF (1 mL) for analysis by proton magnetic resonance spectroscopy (^1^H-MRS) analysis were lyophilized and then redissolved in 500 *μ*L D_2_O, containing 600 *μ*mol/L TSP (sodium trimethylsilylpropionate-2,2,3,3-d_4_) as an internal quantification standard and chemical shift reference. The sample pH was adjusted to 7.0 and centrifuged at 2100 g for 5 min and the supernatant was transferred to 5 mm MRS tubes.

^1^H MR spectra were obtained on a Bruker AVANCE DRX 600 spectrometer (14.1 T, Bruker BioSpin GmbH, Germany), operating at 600.13 MHz for protons. Single pulse proton spectra were obtained at 298 K. One hundred and ninety-two transients were collected into 64 K data points, covering a spectral range of 7.2 kHz. Fully relaxed spectra obtained with a pulse angle of 45°, acquisition time of 4.56 s, and additional repetition delay of 8 s allowed for absolute quantification. The repetition delay included water presaturation during the last 3 s. Spectra were apodized with an exponential line-broadening of 0.3 Hz and Fourier transformed. Metabolite concentrations were obtained by integrating the relevant peaks and relating the areas to the TSP signal at 0 ppm. For peak identification purposes, two-dimensional homonuclear chemical shift correlated (COSY) and J-resolved (JRES) spectra were obtained, together with reference to the literature [20–22]. Due to overlapping signals (shown by JRES spectroscopy to be singlets), the alanine concentration was calculated by curve-fitting (PeakFit version 4.12, Alfasoft AS, Norway). All MRS procedures, including the metabolite quantification, were performed by an investigator unaware of the group to which samples belonged.

Results are expressed as the mean ± SD. Statistical analysis was carried out using SPSS version 19.0, applying Student's *t*-test (5 comparisons: control group with the other 3 groups, as well as a comparison between the groups with PD, and between the two groups with the* LRRK2* p.G2019S mutation), with Levene's test for equality of variances. A Bonferroni correction was applied, whereby values of *p* < 0.01 were considered significant, and values of *p* < 0.05 as a trend. Age and LEDD were tested as possible confounders (ANCOVA). Partial least squares discriminant analysis (PLS-DA) [23] uses both the differences between metabolites and interactions between them to establish levels of statistical separation. PLS-DA was used to model the relationship between the metabolite concentrations and group membership in a supervised manner. The loadings are coloured according to the variable importance in the projection (VIP) scores, positively reflecting the variable's importance for classification [24]. The metabolite concentrations were autoscaled prior to modelling. Cross-validation was performed in order to validate the results. For subsets with less than 30 samples, leave-one-out cross-validation was performed, whereas larger subsets of data were validated by 10-fold cross-validation with 10 repetitions. Leave-one-out cross-validation is more prone to overfitting data than repeated 10-fold cross-validation. However, the significance of the resulting classification performance was evaluated by permutation testing (*n* = 1000) [25], and only values of *p* < 0.05 were considered significant. PLS-DA was performed in Matlab R2009a (The Mathworks, Inc.) using PLS_Toolbox 6.2.1 (Eigenvector Research, Inc.). Pearson's correlation coefficient was calculated to assess correlations between the metabolites. All correlations with values of *p* < 0.05 were confirmed as having a linear distribution (not shown).

## 3. Results

**Figure 1:**
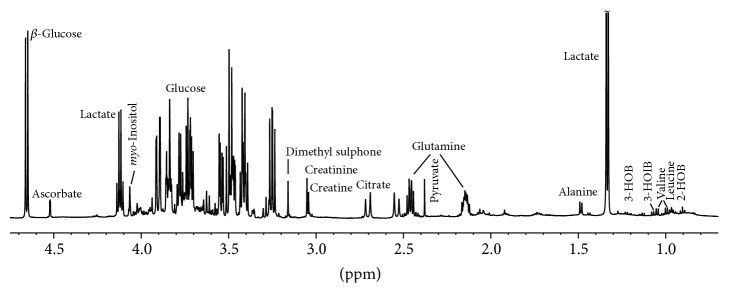
Proton magnetic resonance (^1^H-MR) spectrum of cerebrospinal fluid from a* LRRK2* p.G2019S-PD patient (HOB: hydroxybutyrate, HOIB: hydroxyisobutyrate).

Peak assignments in a ^1^H MR spectrum of a* LRRK2*-PD patient are shown in Figure 1. Intermediary metabolites present in the ^1^H MR spectra, including energy sources and metabolites, several neutral amino acids, three isomers of hydroxybutyrate, and dimethyl sulphone (methylsulfonylmethane, DMSO_2_), were reliably quantified. Although other compounds were identified, integration of these was not performed due to overlapping peaks, or underlying unknown signal patterns.

Metabolite concentrations for patients with* LRRK2*-PD, sporadic PD, and healthy mutation carriers and controls as measured by ^1^H-MRS are shown in Table 3. Analysis with ANCOVA indicated that LEDD had no significant effect on the levels of any metabolite, and only creatinine was dependent on age, as previously demonstrated [26]. However, since no differences in creatinine concentration overall were found and the substance did not contribute particularly to the PLS-DA separations, it is not considered further in this study. No significant differences for any metabolite were found between the control and asymptomatic* LRRK2* groups. Between the sporadic and* LRRK2*-PD groups, only a significant reduction in glucose when measured by MRS was found. This reduction as measured by MRS was also significant when the* LRRK2*-PD group was compared to the control group. However, no significant differences were observed between the groups when total glucose was analysed routinely.

Significant reductions in 2-hydroxybutyrate were found in the* LRRK2*-PD group relative to both the control and healthy mutation carrier groups, as well as a trend between the control and sporadic PD group (*p* = 0.02). However, only trends were found for the level of 3-hydroxybutyrate between the* LRRK2*-PD group and the control (*p* = 0.019) or asymptomatic* LRRK2* group (*p* = 0.015). Similarly, a trend was seen for a reduced level of 3-hydroxyisobutyrate between the* LRRK2*-PD group and the asymptomatic* LRRK2* group (*p* = 0.035).

A trend towards a reduced lactate level (*p* = 0.031) and increased level of ascorbate (*p* = 0.026) was observed between the sporadic PD and the control group.

DMSO_2_ was significantly increased in* LRRK2*-PD relative to the control group, though trends were also found relative to the asymptomatic mutation carriers (*p* = 0.012) and sporadic PD (*p* = 0.017) groups.

Four neutral amino acids were reliably quantified. Glutamine was significantly increased in both groups with PD relative to the control group and correlated well with the other three amino acids in most groups. The concentrations of alanine, valine, and leucine were similar in all groups and correlated with each other in all four groups (*r* = 0.58–0.99, all *p* ≤ 0.015).

**Figure 2:**
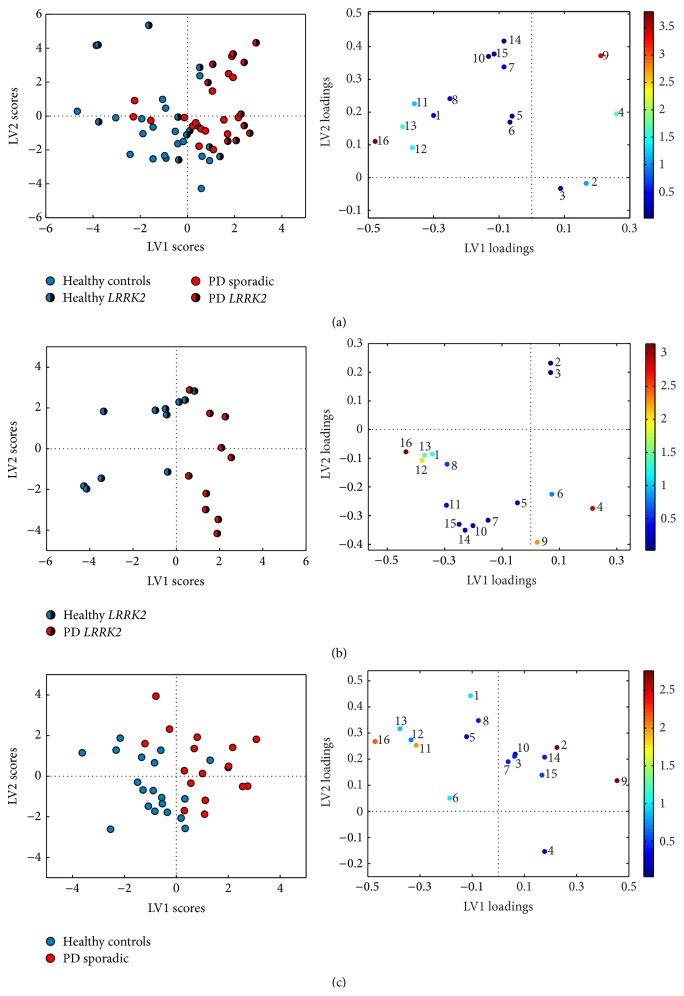
PLS-DA scores and loadings of LV1 and LV2 separating the metabolic profiles of (a) healthy and Parkinson's disease (all patients), (b) healthy* LRRK2* carriers and Parkinson's disease with* LRRK2*, and (c) healthy controls and sporadic Parkinson's disease. Metabolite numbers: (1) *β*-D-glucose, (2) ascorbate, (3)* myo*-inositol, (4) dimethyl sulphone, (5) creatinine, (6) creatine, (7) citrate, (8) pyruvate, (9) glutamine, (10) alanine, (11) lactate, (12) 3-hydroxybutyrate, (13) 3-hydroxyisobutyrate, (14) valine, (15) leucine, and (16) 2-hydroxybutyrate.

The PLS-DA classification results are shown in Table 4. Permutation testing showed significant classification results for all comparisons except for those between the healthy carriers of* LRRK2* p.G2019S and individuals in the control group. The best separation was found between control individuals and* LRRK2*-PD patients (92.4% correct classification). The difference between the two PD groups was only weakly significant at the 5% level. When comparing all healthy individuals against all patients with PD (Figure 2(a)), the loading plot shows that lower levels of all three hydroxybutyrates, together with higher CSF levels of DMSO_2_ and glutamine, were most responsible for driving this separation. Similarly, good separation (85.9% correct classification, Figure 2(b)) was obtained when comparing* LRRK2*-PD patients with healthy mutation carriers, this being largely driven by the same metabolites as in Figure 2(a). The separation obtained between the sporadic PD and the group of healthy control individuals (Figure 2(c)) was not quite so successful (76.6% correct classification). Higher levels of ascorbate and glutamine and lower levels of lactate and 2-hydroxybutyrate, and to a lesser extent 3-hydroxybutyrate and 3-hydroxyisobutyrate, were the most important variables for separating the sporadic PD group from the control group.

**Figure 3:**
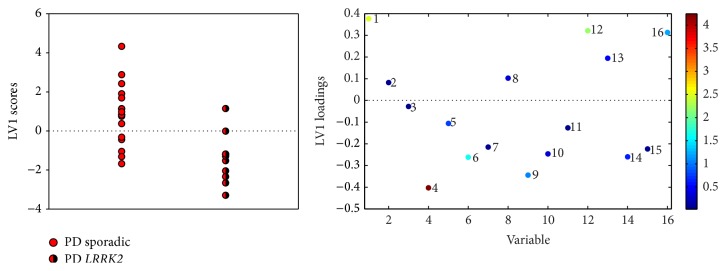
PLS-DA scores and loadings of LV1 separating Parkinson's disease patients with* LRRK2* mutations from patients with sporadic disease. Metabolite numbers: (1) *β*-D-glucose, (2) ascorbate, (3)* myo*-inositol, (4) dimethyl sulphone, (5) creatinine, (6) creatine, (7) citrate, (8) pyruvate, (9) glutamine, (10) alanine, (11) lactate, (12) 3-hydroxybutyrate, (13) 3-hydroxyisobutyrate, (14) valine, (15) leucine, and (16) 2-hydroxybutyrate.

Sporadic PD could be partially separated from* LRRK2*-PD (70.3% correct classification, Figure 3) based on higher levels of glucose, as well as 2- and 3-hydroxybutyrate, and lower levels of DMSO_2_ and glutamine in sporadic PD.

## 4. Discussion

PLS-DA significantly distinguished patients with PD from healthy individuals based on ^1^H-MRS spectroscopy of metabolites in CSF. The best separation was found between the healthy control group and patients with* LRRK2*-PD, though the separation between healthy mutation carriers and* LRRK2*-PD was also very good considering the low number of individuals in each group. The control and sporadic PD groups were distinguished from each other fairly well, though not as successfully as seen with* LRRK2*-PD, which is probably a purer form of the disease in having a common etiology. However, there was some overlap between the asymptomatic and the affected* LRRK2* groups. It has been demonstrated that changes in substantia nigra and dopamine turnover can be detected prior to the onset of motor symptoms [6, 27], and it is possible that those healthy mutation carriers overlapping with the* LRRK2*-PD group in the present study have an ongoing disease process. Sporadic PD could be distinguished from* LRRK2*-PD, though the significance was weaker suggesting that PD with varying etiology might have some distinct pathophysiological mechanisms reflected in different metabolic fingerprints.

One of the main findings of this study was the reduced level of 2-hydroxybutyrate (*α*-hydroxybutyrate) in CSF from patients with* LRRK2*-PD compared to the control and healthy mutation carrier groups, with a similar trend between the sporadic PD and control groups. It was one of the substances most responsible for distinguishing the groups by PLS-DA, especially* LRRK2*-PD from either control group. 2-Hydroxybutyrate is formed from 2-oxobutyrate (*α*-ketobutyrate) by the action of lactate dehydrogenase (LDH). 2-Oxobutyrate can be formed either directly through catabolism of threonine, or through methionine metabolism via homocysteine and cystathionine, where the latter is cleaved to 2-oxobutyrate and cysteine. Cysteine can be incorporated into reduced glutathione (GSH), the main cellular antioxidant providing protection against oxidative stress. There is ample evidence that oxidative stress and mitochondrial energy metabolism are compromised in PD [28]. Moreover, GSH has been found to be reduced in substantia nigra [29, 30]. A reduction in CSF 2-hydroxybutyrate in relation to PD in the present study therefore complements such data as it infers that the reaction might also reduce the level of cysteine available for GSH synthesis (or alternatively the reaction with LDH is reversed to maximise 2-oxobutyrate for cysteine production, thus depleting 2-hydroxybutyrate). Further support for this is provided by our data showing that there is a reduction in the level of GSH measured in isolated peripheral neutrophils (polymorphonuclear leucocytes) from patients with sporadic PD compared to cells from controls (Falkenberg et al. unpublished). Speculatively, therefore, alterations in GSH metabolism may account for the reduction of 2-hydroxybutyrate in PD.

Glutamine was found to be significantly increased in both groups of patients with PD in the present study and contributed to the separation with their respective control group by PLS-DA. Increased glutamine in CSF might reflect increased metabolic activity in the brain generally due to PD pathophysiology, as release of glutamine has been suggested as a means of removing excess nitrogen from brain during disease [31].

Glucose measured by ^1^H-MRS in CSF was lower in the* LRRK2*-PD group compared to other groups and this difference contributed to separation of the PD groups from each other as well as healthy mutation carriers from* LRRK2*-PD. It remains unclear however whether glucose is consistently lower in the CSF of patients with* LRRK2*-PD (p.G2019S) and if so whether this also reflects a change in brain energy metabolism in this condition.

One of the metabolites driving the separation of the control group from sporadic PD using PLS-DA was ascorbate. The concentration of ascorbate in CSF was not significantly increased relative to controls in any group when testing single metabolites, though the level was consistently higher (all *p* < 0.07). Since PLS-DA assesses the relationship between metabolites for separation, ascorbate can be important for PLS-DA analysis without necessarily being important in a univariate analysis. As ascorbate is an essential dietary supplement, as well as an antioxidant, it is perhaps not surprising that many individuals, particularly those with a disease, ensure they take an adequate amount. Unfortunately we have no data regarding vitamin use, but the contribution of ascorbate towards the PLS-DA separation need not be related to the pathological process.

It is interesting that DMSO_2_ was significantly increased in* LRRK2*-PD, though not sporadic PD. An earlier ^1^H-MRS study [32] found significantly increased amounts of an unknown factor in CSF, later shown to be DMSO_2_ [20], in connection with several forms of dementia. No significant increase was seen in CSF from patients with idiopathic PD, agreeing with the results from sporadic PD in the present study. However, it is tempting to infer a role for DMSO_2_ in connection with certain neurodegenerative diseases. Such a function is uncertain as DMSO_2_ not only is a dietary substance, but also can be taken as a health product even though it has no proven medical benefit. Nevertheless, it is thought that (unlike ascorbate) it can be derived endogenously and that dietary sources alone cannot usually account for its concentration in plasma and CSF [20]. DMSO_2_ has the ability to cross membranes, including the blood-brain barrier, and has anti-inflammatory activity, suppressing the production of active radicals by activated neutrophils in vitro [33]. It has not shown marked neurochemical toxicity by in vivo ^1^H-MRS [34]. The increase in connection with* LRRK2*-PD and other neurodegenerative diseases might be a response to increased production of active radicals in connection with the disease process and thus a protective mechanism against neuronal loss.

One interesting substance that could not be included in the present study was hypoxanthine, which a previous study of metabolic profiling of plasma samples using LCECA showed to be significantly decreased in patients with PD, as were the ratios of major metabolites of the purine pathway [12]. This suggested possible aberrations upstream of uric acid in PD. Unfortunately, hypoxanthine occurs in CSF at a concentration which is on the border of what can be reliably quantitated (low signal-to-noise ratio) with the 600 MHz/14.1 Tesla spectrometer.

Anti-Parkinson medication is well known to affect regional cerebral glucose metabolism [35, 36] and may therefore alter metabolic balance in general. Although dopamine-replacement therapy levels were adjusted to a levodopa equivalent daily dose [18] and no significant correlations were found between the various metabolites and drug dose, it is still unknown to what extent the changes in the PD groups were attributable to therapy. This could only have been found by analysing results from patients temporarily off drugs. The groups in the present study are small, so conclusions must be drawn with care. However, L-DOPA competes with neutral amino acids for the same transporter system for entry into brain [37], but in the present material the neutral amino acids alanine, valine, and leucine were all strongly correlated in all groups and there were no significant differences in concentration. This suggests that L-DOPA did not interfere with the transport of these amino acids over the blood-brain barrier. Even glutamine correlated in most cases with the other amino acids measured, suggesting that the increases seen in PD are based on the disease state rather than the therapy. Taken together, these results support the reliability of the results overall.

Advanced laboratory techniques for metabolomics can identify hundreds of metabolites but are not yet generally available. ^1^H-MRS has more practical potential. Samples prepared for ^1^H-MRS can be used over time if necessary, which can be an advantage for samples like CSF that are not as easily available as blood. The range of the method may be limited at present, but, as the technique improves to measure metabolites at lower concentrations, it should offer a more readily available metabolomics method with sufficient metabolites to distinguish disease from control groups.

## 5. Conclusions

Under our conditions, ^1^H-MRS gave reliable results for 16 intermediary metabolites in CSF, and PLS-DA distinguished PD patients from healthy individuals, with the best results being obtained between genetic PD and control groups. 2-Hydroxybutyrate, glutamine, and dimethyl sulphone largely contributed to the separations, perhaps altering concentration in response to biochemical processes related to PD pathology; glutamine as a means of removing excess nitrogen from brain, dimethyl sulphone as an anti-inflammatory agent, and 2-hydroxybutyrate in connection with altered glutathione metabolism. As MR-techniques improve, ^1^H-MRS can be a promising tool for identifying novel biomarkers for PD.

## Figures and Tables

**Table 1:** Demographic data of the study participants.

	Sporadic PD	*LRRK2*-PD	Asymptomatic *LRRK2 *carriers	Control
*N*	17	10	11	19
Age	61.9 ± 7.6	70.4 ± 11.7^a^	63.4 ± 11.3	57.7 ± 8.3
Sex (male/female)	13/4	6/4	5/6	10/9
CSF free cells (10^6^/L)	1.5 ± 1.1	1.2 ± 1.1	2.0 ± 1.1	2.2 ± 1.4
CSF total protein (g/L)	0.45 ± 0.24	0.50 ± 0.24	0.46 ± 0.12	0.40 ± 0.10
Age at disease onset	54.7 ± 10.5	58.5 ± 10.3		
Duration of disease (y)	8.0 ± 5.9	11.9 ± 7.1		
Hoehn and Yahr	2.5 ± 0.98	2.6 ± 0.96		
Levodopa/carbidopa (mg/day)	403 ± 295	385 ± 111		
Levodopa (equivalent daily dose, LEDD)	529 ± 321	515 ± 233		

Results are given as the mean ± SD. PD = Parkinson's disease. There were no significant differences between the groups for CSF free cells or total protein (nor between the two groups with PD for levodopa or LEDD). ^a^There was a significant difference in age between the control and *LRRK2*-PD groups, *p* = 0.002.

**Table 2:** Anti-Parkinson agents used for the treatment of the two patient groups.

Patient number	PD group	Treatment
1	*LRRK2*-PD	Carbidopa/levodopa 300 mg
2		Carbidopa/levodopa 300 mg
3		Carbidopa/levodopa 500 mg, pramipexole 0.7 mg ×3
4		Carbidopa/levodopa 400 mg, pergolide 1 mg ×3
5		Carbidopa/levodopa 500 mg, pramipexole 0.7 mg ×3
6		Carbidopa/levodopa 400 mg
7		Carbidopa/levodopa 200 mg, STN DBS
8		Carbidopa/levodopa 550 mg, pramipexole 0.7 mg ×3
9		Carbidopa/levodopa 300 mg
10		Carbidopa/levodopa 400 mg, ropinirole 18 mg, selegiline 10 mg

11	Sporadic PD	Carbidopa/levodopa 1000 mg
12		Carbidopa/levodopa 1000 mg, pramipexole 0.7 mg ×3
13		Carbidopa/levodopa 300 mg, ropinirole 20 mg
14		Carbidopa/levodopa 300 mg
15		Cabergoline 3 mg
16		Carbidopa/levodopa 400 mg, pramipexole 0.18 mg ×3
17		Carbidopa/levodopa 600 mg, pramipexole 0.7 mg ×3
18		Carbidopa/levodopa 400 mg
19		Carbidopa/levodopa 800 mg, pramipexole 0.7 mg ×4
20		Carbidopa/levodopa 400 mg, pramipexole 0.36 mg ×3, selegiline 10 mg
21		Carbidopa/levodopa 150 mg, cabergoline 3 mg
22		Carbidopa/levodopa 300 mg, STN DBS
23		Carbidopa/levodopa 300 mg
24		Carbidopa/levodopa 300 mg, rotigotine 12 mg
25		Carbidopa/levodopa 400 mg, selegiline 10 mg
26		Carbidopa/levodopa 200 mg, STN DBS
27		Pramipexole 0.36 mg ×3, selegiline 10 mg

Patient number 19 also took an antidepressant, escitalopram 10 mg. Selegiline is a monoamine oxidase-B inhibitor. STN DBS = deep brain stimulation of the subthalamic nucleus.

**Table 3:** Intermediary metabolite levels in cerebrospinal fluid measured by proton magnetic resonance spectroscopy.

Metabolite	Chemical shift (ppm)	Sporadic PD (*n* = 17)	*LRRK2*-PD (*n* = 10)	Asymptomatic *LRRK2* p.G2019S (*n* = 11)	Control (*n* = 19)
*β*-D-Glucose	4.66	2240 ± 357	1973 ± 81^a,b^	2296 ± 482	2177 ± 233
*Glucose* (mM)	na	*3.9* ± *1.2*	*3.4* ± *0.7*	*3.9* ± *0.8*	*3.7* ± *0.4*
Pyruvate	2.38	46.0 ± 8.1	42.7 ± 13.3	51.8 ± 17.9	44.6 ± 12.3
Lactate	1.33	1703 ± 142	1717 ± 219	1830 ± 382	1860 ± 261
Citrate	2.53–2.72	262 ± 52	267 ± 47	270 ± 72	250 ± 47
Creatine	3.04	48.9 ± 4.1	54.3 ± 11.2	49.7 ± 3.3	52.0 ± 7.7
Glutamine	2.46	528 ± 66^d^	567 ± 68^c^	508 ± 67	466 ± 66
Alanine	1.48	45.0 ± 11.5	49.0 ± 14.6	51.9 ± 19.4	44.1 ± 11.0
Valine	1.05	16.9 ± 5.3	19.6 ± 8.6	21.5 ± 8.9	15.4 ± 3.5
Leucine	0.97	14.3 ± 4.4	15.3 ± 6.4	17.3 ± 6.6	12.8 ± 3.3
2-Hydroxybutyrate	0.90	22.5 ± 9.7	17.4 ± 4.9^e,f^	33.8 ± 12.6	31.8 ± 12.7
3-Hydroxybutyrate	1.20	8.2 ± 4.4	5.2 ± 1.8	12.8 ± 8.6	9.8 ± 7.2
3-Hydroxyisobutyrate	1.07	11.5 ± 2.1	10.7 ± 2.5	14.8 ± 5.2	12.5 ± 2.2
*myo*-Inositol	4.07	131 ± 27	135 ± 35	135 ± 38	123 ± 26
Dimethyl sulphone	3.16	9.1 ± 4.9	17.7 ± 9.1^g^	8.3 ± 4.7	8.2 ± 3.3
Creatinine	3.05	68.4 ± 13.2	75.7 ± 18.0	73.4 ± 14.9	68.4 ± 10.3
Ascorbate	4.52	165.7 ± 35.2	171.6 ± 55.3	166.9 ± 30.7	137.2 ± 38.0

Concentrations (mean ± SD) are given in *μ*mol/L, except for glucose analysed routinely by the University Hospital, which is given in mM (in italics for comparison). The *β*-anomer of D-glucose was significantly reduced in the *LRRK2*-PD group relative to the control group (^a^ *p* = 0.002), and the sporadic PD group (^b^ *p* = 0.008). Glutamine was significantly increased in both the *LRRK2*-PD (^c^ *p* = 0.001) and the sporadic PD (^d^ *p* = 0.009) groups relative to the control group. Significant reductions in 2-hydroxybutyrate were found in the *LRRK2*-PD group relative to both the control (^e^ *p* < 0.0005) and the asymptomatic *LRRK2* p.G2019S (^f^ *p* = 0.001) groups. Dimethyl sulphone was significantly increased in the *LRRK2*-PD group relative to the control group (^g^ *p* = 0.009).

**Table 4:** Classification results from PLS-DA of intermediary metabolite levels in cerebrospinal fluid measured by proton magnetic resonance spectroscopy.

Comparisons	*N*	Correct classification (%)	Sensitivity (%)	Specificity (%)	LVs	*p* value
All PD versus all healthy individuals	57	74.2	74.7	73.7	2^*∗*^	0.001
*LRRK2*-PD versus controls	29	92.4	90.0	94.7	2^*∗∗*^	<0.001
*LRRK2*-PD versus asymptomatic mutation carriers	21	85.9	81.8	90.0	2^*∗∗*^	0.001
Sporadic PD versus controls	36	76.6	70.6	82.6	2^*∗*^	0.002
Sporadic PD versus *LRRK2*-PD	27	70.3	70.6	70.0	1^*∗∗*^	0.038
Asymptomatic mutation carriers versus controls	30	64.1	54.5	73.7	1^*∗∗*^	0.103

Asymptomatic mutation carriers: healthy individuals carrying the *LRRK2* p.G2019S mutation; LVs: latent variables; ^*∗*^random subsets, 10 divisions and 10 repetitions. ^*∗∗*^Leave-one-out cross-validation.

## References

[1] Fahn S. (2003). Description of Parkinson's disease as a clinical syndrome. *Annals of the New York Academy of Sciences*.

[2] Singleton A. B., Farrer M. J., Bonifati V. (2013). The genetics of Parkinson's disease: progress and therapeutic implications. *Movement Disorders*.

[3] Trinh J., Farrer M. (2013). Advances in the genetics of Parkinson disease. *Nature Reviews Neurology*.

[4] Mata I. F., Kachergus J. M., Taylor J. P. (2005). Lrrk2 pathogenic substitutions in Parkinson's disease. *Neurogenetics*.

[5] Aasly J. O., Toft M., Fernandez-Mata I. (2005). Clinical features of *LRRK2*-associated Parkinson's disease in central Norway. *Annals of Neurology*.

[6] Sossi V., de La Fuente-Fernández R., Nandhagopal R. (2010). Dopamine turnover increases in asymptomatic *LRRK2* mutations carriers. *Movement Disorders*.

[7] Aasly J. O., Shi M., Sossi V. (2012). Cerebrospinal fluid amyloid *β* and tau in *LRRK2* mutation carriers. *Neurology*.

[8] Aasly J. O., Johansen K. K., Brønstad G. (2014). Elevated levels of cerebrospinal fluid *α*-synuclein oligomers in healthy asymptomatic *LRRK2* mutation carriers. *Frontiers in Aging Neuroscience*.

[9] Shi M., Bradner J., Hancock A. M. (2011). Cerebrospinal fluid biomarkers for Parkinson disease diagnosis and progression. *Annals of Neurology*.

[10] Jimenez-Jimenez F. J., Alonso-Navarro H., Garcia-Martin E., Agundez J. A. (2014). Cerebrospinal fluid biochemical studies in patients with Parkinson's disease: toward a potential search for biomarkers for this disease. *Frontiers in Cellular Neuroscience*.

[11] Henchcliffe C. (2015). Blood and cerebrospinal fluid markers in Parkinson's disease: current biomarker findings. *Current Biomarker Findings*.

[12] Johansen K. K., Wang L., Aasly J. O. (2009). Metabolomic profiling in *LRRK2*-related Parkinson's disease. *PLoS ONE*.

[13] Trupp M., Jonsson P., Ohrfelt A. (2014). Metabolite and peptide levels in plasma and CSF differentiating healthy controls from patients with newly diagnosed Parkinson's disease. *Journal of Parkinson's Disease*.

[14] Del Bigio M. R. (1995). The ependyma: a protective barrier between brain and cerebrospinal fluid. *Glia*.

[15] Bruni J. E. (1998). Ependymal development, proliferation, and functions: a review. *Microscopy Research and Technique*.

[16] Segal M. B. (2000). The choroid plexuses and the barriers between the blood and the cerebrospinal fluid. *Cellular and Molecular Neurobiology*.

[17] Gelb D. J., Oliver E., Gilman S. (1999). Diagnostic criteria for Parkinson disease. *Archives of Neurology*.

[18] Zibetti M., Pesare M., Cinquepalmi A. (2008). Antiparkinsonian therapy modifications in PD patients after STN DBS: a retrospective observational analysis. *Parkinsonism and Related Disorders*.

[19] Kachergus J., Mata I. F., Hulihan M. (2005). Identification of a novel *LRRK2* mutation linked to autosomal dominant parkinsonism: evidence of a common founder across European populations. *American Journal of Human Genetics*.

[20] Engelke U. F., Tangerman A., Willemsen M. A. (2005). Dimethyl sulfone in human cerebrospinal fluid and blood plasma confirmed by one-dimensional ^1^H and two-dimensional ^1^H-^13^C NMR. *NMR in Biomedicine*.

[21] Lutz N. W., Maillet S., Nicoli F., Viout P., Cozzone P. J. (1998). Further assignment of resonances in ^1^H NMR spectra of cerebrospinal fluid (CSF). *FEBS Letters*.

[22] Lutz N. W., Viola A., Malikova I. (2007). A branched-chain organic acid linked to multiple sclerosis: first identification by NMR spectroscopy of CSF. *Biochemical and Biophysical Research Communications*.

[23] Wold S., Sjöström M., Eriksson L. (2001). PLS-regression: a basic tool of chemometrics. *Chemometrics and Intelligent Laboratory Systems*.

[24] Chong I.-G., Jun C.-H. (2005). Performance of some variable selection methods when multicollinearity is present. *Chemometrics and Intelligent Laboratory Systems*.

[25] Westerhuis J. A., Hoefsloot H. C. J., Smit S. (2008). Assessment of PLSDA cross validation. *Metabolomics*.

[26] Niklasson F., Agren H. (1984). Brain energy metabolism and blood-brain barrier permeability in depressive patients: analyses of creatine, creatinine, urate, and albumin in CSF and blood. *Biological Psychiatry*.

[27] Sierra M., Sánchez-Juan P., Martínez-Rodríguez M. I. (2013). Olfaction and imaging biomarkers in premotor *LRRK2* G2019S-associated Parkinson disease. *Neurology*.

[28] Kumar H., Lim H.-W., More S. V. (2012). The role of free radicals in the aging brain and Parkinson's disease: convergence and parallelism. *International Journal of Molecular Sciences*.

[29] Sian J., Dexter D. T., Lees A. J. (1994). Alterations in glutathione levels in Parkinson's disease and other neurodegenerative disorders affecting basal ganglia. *Annals of Neurology*.

[30] Sofic E., Lange K. W., Jellinger K., Riederer P. (1992). Reduced and oxidized glutathione in the substantia nigra of patients with Parkinson's disease. *Neuroscience Letters*.

[31] Grill V., Björkman O., Gutniak M., Lindqvist M. (1992). Brain uptake and release of amino acids in nondiabetic and insulin-dependent diabetic subjects: important role of glutamine release for nitrogen balance. *Metabolism*.

[32] White L. R., Gårseth M., Aasly J., Sonnewald U. (2004). Cerebrospinal fluid from patients with dementia contains increased amounts of an unknown factor. *Journal of Neuroscience Research*.

[33] Beilke M. A., Collins-Lech C., Sohnle P. G. (1987). Effects of dimethyl sulfoxide on the oxidative function of human neutrophils. *The Journal of Laboratory and Clinical Medicine*.

[34] Lin A., Nguy C. H., Shic F., Ross B. D. (2001). Accumulation of methylsulfonylmethane in the human brain: identification by multinuclear magnetic resonance spectroscopy. *Toxicology Letters*.

[35] Berding G., Odin P., Brooks D. J. (2001). Resting regional cerebral glucose metabolism in advanced Parkinson's disease studied in the *off* and *on* conditions with [^18^F]FDG-PET. *Movement Disorders*.

[36] Klein R. C., de Jong B. M., de Vries J. J., Leenders K. L. (2005). Direct comparison between regional cerebral metabolism in progressive supranuclear palsy and Parkinson's disease. *Movement Disorders*.

[37] del Amo E. M., Urtti A., Yliperttula M. (2008). Pharmacokinetic role of L-type amino acid transporters LAT1 and LAT2. *European Journal of Pharmaceutical Sciences*.

